# Authors' Response to Peer Reviews of “COVID-19 and Cybersecurity: Finally, an Opportunity to Disrupt?”

**DOI:** 10.2196/29517

**Published:** 2021-05-06

**Authors:** Ana Ferreira, Ricardo Cruz-Correia

**Affiliations:** 1 CINTESIS Faculty of Medicine University of Porto Porto Portugal; 2 MEDCIDS Faculty of Medicine University of Porto Porto Portugal

**Keywords:** COVID-19, cybersecurity, challenges and disruption, data protection, privacy, health data

*This is the authors' response to peer-review reports for "COVID-19 and Cybersecurity: Finally, an Opportunity to Disrupt?"*

## Round 1 Review:

### Reviewer B:

#### General Comments

Thank you very much for your comment [1]. In this case, the paper [2] is a viewpoint and not an original research article. Its main objective is to raise important cybersecurity issues for further discussion and improvement that had not yet been raised in the research literature (as of May 2020). Therefore, there are no new identified issues or results to provide at this stage, but we provide more awareness and the ability to reach different stakeholders and the population in general. Indeed, no new or novel cybersecurity issues were found, but we discuss the exacerbation of already existing issues due to the pandemic, which do not yet have proper solutions. Finally, as mentioned in the submission, this was a summarized version of another research paper (at the time still under review) which has since been accepted for publication in conference proceedings [3]. This full research paper, which obtained the Best Paper Award, was also added to the References section of the viewpoint for more details and completeness. Also, a new sentence (“More details on the background research that was performed as a basis to this viewpoint can be found here [3].”) was added at the end of the introduction to make readers aware early in their reading that more details can be found in that reference.

#### Specific Comments

##### Major Comments

1. Thank you very much for your comment. Due to the aim of this viewpoint, it was not possible to further extend the discussion to all the presented subjects. However, the authors opted to focus on the message they wanted to give in all of them. In the case of contact tracing apps, the main point the authors wanted to make is that there are still many security and privacy issues that are not yet well defined and understood. These need to be addressed first in order to ensure that these apps can be used in the most private, secure, and useful manner. Comparing and discussing how various countries and cultures worldwide are adopting this technology and adapting it to their reality was not a goal of this viewpoint, although the authors see how interesting and relevant this topic is. Nevertheless, this could also enter the realm of ethical, political, social, cultural, and humanitarian-related subjects, which could not be well addressed in this very short and general document.
2. Thank you very much for your comment. As mentioned in the previous answer, the authors could not discuss every single cybersecurity issue in this viewpoint. Furthermore, the subject you refer to does not seem to be directly related to the aim of the document or to COVID-19.

##### Minor Comments

Thank you very much for your comment. Corrections have been made accordingly.

### Anonymous [4]

#### General Comments

1. Thank you very much for this comment [4]. The editor’s comments and requirements will certainly be taken into account.
2. Thank you very much for this comment. In fact, with the aim to provide the most recent references, and to focus on a broad topic such as cybersecurity in a viewpoint, it was not possible to refer to many technical aspects because this would not be feasible in such a short document. However, in the revised version of this viewpoint, there are at least 8 references (24%) that focus on the most common issue on the literature at the time (May 2020)—contact tracing apps—which are certainly related more to technical security and privacy content. Moreover, as mentioned in the submission, this viewpoint is a summarized version of another research paper (at the time still under review), which was then accepted for publication in conference proceedings [3]. In this full research paper, a literature review returned only 18 scientific documents, of which only 10 were full articles that aimed to discuss challenges and concerns related to cybersecurity and COVID-19 at the time (May 2020). The paper, which received the Best Paper Award, was also added to the reference list of this viewpoint to provide more details and completeness. A new sentence (“More details on the background research that was performed as a basis to this viewpoint can be found here [3].”) was also added at the end of the Introduction section to quickly refer interested readers to more details. In conclusion, this viewpoint was written with the goal to simply and quickly reach a variety of stakeholders (in multidisciplinary fields) as well as the general population, so its content could not be too specific or too technical. Finally, no new or novel cybersecurity issues were found, but we discussed the exacerbation of already existing issues due to the pandemic, which do not yet have proper solutions.

#### Specific Comments

##### Major Comments

1. Thank you very much for this comment. At the moment when this viewpoint was written (May 2020), there were still few studies available in the literature that focused on the cybersecurity issues closely associated with or exacerbated by the pandemic, and those that existed focused on specific issues such as contact tracing apps and the issues involved in sharing data to further research and understand the evolution of the pandemic; however, very few studies addressed other issues that were more consequential or indirect challenges that were more hidden, such as physical security, remote cybersecurity, or the increase of social engineering attacks. Although these topics were at times mentioned in the news/media, there was no comprehensive list of the many issues involved or any attempt to categorize them. With a keen interest in cybersecurity, the authors realized that such a viewpoint, submitted to a well-reputed and well-known scientific journal, could be a useful means to reach a wider audience, not only in cybersecurity and health care but also in other areas. The goal was not to send a “new message” but to bring to light more hidden issues that were not yet thought of but that still need to be stressed, with the aim of developing new, quick, and innovative solutions (eg, theft and fraud, physical security and cybersecurity, and privacy vulnerabilities and threats from working from home/homeschooling). Indeed, no new or novel cybersecurity issues were found, but we discussed the exacerbation of existing issues due to the pandemic, which do not yet have proper solutions.
2. Thank you very much for this comment. In the impossibility to provide an extended discussion on this relevant issue, a new phrase was added in the “Data Sharing” section, together with two references to support this subject: “Further, the lack of IT and cybersecurity literacy may make it harder for most individuals to adequately install and use those apps, which may, just by themselves, integrate several security vulnerabilities and risks [5][6].”
3. Thank you very much for your comment. Due to the aim of this viewpoint, it was not possible to further extend discussion on the presented subjects. However, the authors opted to focus on the message they wanted to give in all of them. In the case of contact tracing apps, the main point the authors wanted to make was that there are still many security and privacy issues that are not yet well defined and understood. These issues need to be addressed first in order to ensure those apps can be used in the most private, secure, and useful manner. Comparing and discussing how various countries and cultures worldwide are adopting this technology and adapting it to their reality was not a goal of this viewpoint, although the authors see how interesting and relevant this topic is. Nevertheless, this can enter into the realm of ethical, political, social, cultural, and humanitarian-related subjects, which could not be well addressed in this very short and broad document. In addition, as the main cybersecurity literature at the time (May 2020) focused mostly on contact tracing apps, the authors wanted to bring the attention of the research community and the general public to other important cybersecurity and COVID-19–related subjects that were being neglected; therefore, the authors chose to aggregate more relevant issues, but unfortunately without leaving much space for detailing them in a viewpoint.
4. Thank you very much for this comment. Indeed, not all issues were introduced and discussed because this was not an original research paper but was based on another conference paper [3], so not all issues were covered/discussed equally. The authors needed to choose which issues to highlight in this viewpoint. However, the authors have included within the manuscript the following sentence, which highlights a similar issue: “Increased risky behaviours with individuals constantly online in all sorts of activities, using home/personal digital infrastructures and devices (not security-prepared), for different contexts, requirements and goals.” at the end of the paragraph of the section *COVID-19, CYBERSECURITY, PRIVACY, SECURITY AND SAFETY*, which may also raise the issue identified by the reviewer.
5. Thank you very much for this comment. In fact, the authors referred to this very relevant issue in the viewpoint (in the section *COVID-19 AND CYBERSECURITY: DIRECT CONSEQUENCES*: “It is also very difficult to control de boundaries of the protocol itself, since many ‘false positive contacts’ can be obtained when Bluetooth and/or GPS signals can traverse walls, cars, etc, where individuals were not really in contact with someone infected, but may have walked past on the other side of those boundaries”), although not much detailed discussion was provided. As such, the authors felt it was relevant to complement this mention with additional text and references [7-9]:
  “i) Bluetooth and/or GPS signals can traverse walls, cars, where individuals were not really in contact with someone infected, but may have walked past on the other side of those boundaries. In this case, the distance estimation may also change depending on the type or smartphone brand/protocols that are used as well as the environmental characteristics and context where they may be communicating with each other. There may be the need to adjust the way this is done according to the pandemic evolution. For instance, it may be safer to have higher false positives than high false negatives, especially if the virus is very contagious [7];
  ii) real people misreport their symptoms;
  iii) spoofing attacks of GPS coordinates to an app are very easily performed in apps with no authentication mechanisms [8].”
6. Thank you very much for this comment. Because it is impossible to provide an extended discussion on this relevant issue, a new phrase was added in the *Data Sharing* section, together with two references to support this subject:
  “Further, the lack of IT and cybersecurity literacy may make it harder for most individuals to adequately install and use those apps, which may, just by themselves, integrate several security vulnerabilities and risks [5][6].”
7. Thank you very much for this comment. When the authors wrote and submitted this viewpoint, very few references on this issue were available (May 2020). Due also to other reviewers’ comments, several references were added and some replaced (references already used in the viewpoint were repeated in places where they were needed—the first paragraph of the *Data Sharing* section). References [10,11] were replaced, and references [7-9] were added.
8. Thank you very much for this comment. Due to the very insightful reviews of this viewpoint, it is obvious that it needs to be clear that this research document is a viewpoint, with viewpoint characteristics. As such, the authors altered the title of this viewpoint to reflect just that: “COVID-19, Cybersecurity and the Human Right to Privacy: A Viewpoint.”

### Anonymous [12]

#### Minor Comments

1. Thank you very much for this comment [12]. Indeed, the authors agree that the conclusion was too broad, and they have summarized the main points that need awareness and addressing so that at the end of the document, the readers will remain interested and will want to know more about those subjects. The added sentence is the following:

“This viewpoint is a crucial alert for the many cybersecurity issues that need proper and adequate measures, which have not yet had a voice in the existing literature, e.g.: i) research integrity and the need for a balance between anonymity and data quality; ii) hidden privacy and technical and non-technical issues related to contact tracing apps; iii) the pressure or stress in existing vulnerable and underbudgeted or obsolete healthcare infrastructures; iv) the exponential increase of social engineering messages (phishing and ransomware) during the pandemic time and the incapacity or inexistence for proper prevention, detection and recovery solutions; v) the pressure to manage the exacerbation of other health conditions with unsecure home-based virtual infrastructures with unaware patients; and vi) the same issues to deal with, when performing every other daily activities online, with the high exposure of both digital and physical entities with many impacts on privacy, cyber and physical security of the entire world population.”

### Anonymous [13]

1. Thank you very much for this comment [13]. All abbreviations were explained accordingly.
2. Thank you very much for this comment. This was corrected accordingly; because the references in the following sentence were also related to this one, the authors merged the two sentences and thus the references are clearly stated now.
3. Thank you very much for this comment. The authors agree with the reviewer on this subject; however, due to space constraints and the paper being a viewpoint, it is not possible to make detailed discussions on this issue. However, the authors have introduced a last paragraph before the Limitations and the Conclusion section, in which they raise awareness of the fact that cybersecurity technology and solutions may exist but that they do not mean much if the human element (more or less knowledgeable) is not prepared. Training, research, and documentation are examples of ways to improve this knowledge; therefore, the following text and reference were added to the viewpoint:
  “And finally, none of these recommendations will be enough if they are not complemented with objective, useful and accurate procedural data and documentation for the general public to adequately protect themselves. Since the majority of cyber incidents are human enabled there needs to be a shift to research underexplored areas of social and behavioral aspects of cybersecurity, to improve the current situation [14]. Cybersecurity literacy is essential, and even more, during pandemic times. One way to do this is by generating scientific research, such as this viewpoint, to raise awareness, provide recommendations and try new or improved solutions. Furthermore, training and awareness material needs to be personalized because users can have different understandings, experiences, backgrounds, motivations, and so on. The unpredictable nature of human behavior and actions make Human an important element and the main enabler of the level of cybersecurity each system can and will have [14].”
4. Thank you very much for this comment. The authors agree that this subject is very important in pandemic times, when people rely almost exclusively on the digital tools available to communicate. Therefore, the authors performed a query in several research engines but did not obtain much scientific data on how the European Union is addressing this issue; available work focuses more on specific studies from specific countries. Commonly, machine learning methods are being used and tested to improve misinformation detection. However, due to the relevance of this subject, the authors have added a sentence in which this subject is discussed, in the last paragraph on page 6, together with two references:
  “Our representatives and the ones that are responsible to communicate and educate the public through news, media, social networks or similar means (which can quickly exponentiate these type of actions), need to be able to pass the correct message as misinformation and fake news can generate confusion and insecurity among the population [15]. Digital tools associated with the virus should not be used to perform political attacks, donation solicitations, business promotion, stock market advice, animal rights campaigning, bioweapons conspiracy theories, unverifiable claims by politicians or to sell face masks or other materials which may not necessarily protect the wearer [16]. The most important, in the end, is to enforce the message that, although with the appropriate care, security and safety measures, people need to be assisted and treated humanely and correctly, because the loss of one is always the loss of all, either now or in the future.”
5. Thank you very much for this comment. Indeed, the authors agree that the conclusion was too broad; however, as this paper is a viewpoint and not an original article with new results or outcomes, the authors felt it was necessary to restress the main points they want the reader to remember and therefore have summarized them at the end of the document, so as not to be just a repetition of the abstract but also to take into account another reviewer’s suggestion. The added sentence is the following:
  “This viewpoint is a crucial alert for the many cybersecurity issues that need proper and adequate measures, which have not yet had a voice in the existing literature, e.g.: i) research integrity and the need for a balance between anonymity and data quality; ii) hidden privacy and technical and non-technical issues related to contact tracing apps; iii) the pressure or stress in existing vulnerable and underbudgeted or obsolete healthcare infrastructures; iv) the exponential increase of social engineering messages (phishing and ransomware) during the pandemic time and the incapacity or inexistence for proper prevention, detection and recovery solutions; v) the pressure to manage the exacerbation of other health conditions with unsecure home-based virtual infrastructures with unaware patients; and vi) the same issues to deal with, when performing every other daily activities online, with the high exposure of both digital and physical entities with many impacts on privacy, cyber and physical security of the entire world population.”
6. Thank you very much for this comment. The paper has been edited accordingly.
7. Thank you very much for this comment. Although the goal of this viewpoint was not to detail any specific cybersecurity subject, and at the time of performing a review for the article [3] on which this viewpoint is based (May 2020), there were not enough studies in the literature to be able to understand the provided solutions, developments, and so on, the authors have included a limitation sentence to highlight this issue, and they have altered the title of the viewpoint to make sure readers understand that this document is just a summary to raise awareness of the subject and have stressed this in the *Conclusion* section. In fact, that article [3] was also included as a reference in the viewpoint for the possibility of providing more detail on this research to the interested parties.
  "This viewpoint has some limitations. The fact that it is a viewpoint, with space constraints, and based on an original paper published in conference proceedings makes it harder to include more technical and detailed discussion about the introduced subjects and related recommendations. However, the main goal of this viewpoint is to summarize, raise awareness and reflect the opinions of its authors to contribute to a wider acknowledgement of the importance of cybersecurity, how it suddenly affects every human activity and the impact it can have on people’s security, privacy and safety.”
8. Thank you very much for this comment. The authors are very pleased to accept these suggestions and have added the mentioned references as relevant to the viewpoint references section, accordingly, with the numbers written at the end of each suggested reference, when applicable.

1. Added as [17]
2. Added as [18]
3. Added as [19]
4. No major focus on cybersecurity
5. No major focus on cybersecurity
6. Repeated above; added as [18]

### Anonymous [20]

1. Thank you very much for your comment [20]. In this case, this paper is a viewpoint and not an original research article. Its main objective is to raise important cybersecurity issues for further discussion and improvement that had not yet been raised in the research literature (as of May 2020). Therefore, there are no new identified issues or results to provide at this stage, but more awareness and the ability to reach different stakeholders and the population in general. Indeed, no new or novel cybersecurity issues were found, but we discuss the exacerbation of already existing issues due to the pandemic, which do not yet have proper solutions. In addition, material has been scattered in the media and so on, but there was no single document to alert and provide a wider overview and awareness of the many cybersecurity issues directly and indirectly linked to COVID-19. Moreover, as mentioned in the submission, this was a summarized version of another research paper (at the time still under review) which was then accepted for publication [3]. This full research paper, which obtained the Best Paper Award, was also added to the *References* section of the viewpoint to provide more details and completeness. Also, a new sentence, “More details on the background research that was performed as a basis to this viewpoint can be found here [3].” was added at the end of the introduction to make readers aware early in their reading that more details could be found in that reference.
2. Thank you very much for your comment. Please refer to the previous comment and the response, which explains that the knowledge gap with the identification of the current state of the art at that time (May 2020) was identified within a published award-winning article on this subject, on which this viewpoint is based.
3. Thank you very much for this comment. At the moment of writing this viewpoint (May 2020), the scientific work to be found in this area was more limited. As such, it is possible that this work was not yet available or was still in press. However, in order to enrich and complete the referred work, the suggested reference was added as [9].
4. Thank you very much for your comment. In this case, this is a viewpoint and not an original research article, with space and content constraints for the wide subjects that the authors wanted to highlight. Its main objective is to raise important cybersecurity issues for further discussion and improvement that had not yet been raised in the research literature (as of May 2020); therefore, it is more generic and provides more qualitative descriptions. For more quantitative analyses, please refer to the research paper this work is based upon [3], a reference to which is also included in the viewpoint.

## Round 2 Review:

Dear Editor(s), thank you for your comments and suggestions. I have responded below to the reviewers who still have some problems with the manuscript. Please let me know your decision as soon as possible. Kind regards, and thank you for the opportunity.

### External Peer-Review Reports

#### Anonymous [4]

##### General Comments

Thank you very much for your comment. First of all, we want to remind the reviewer that this viewpoint was submitted in early June 2020, when not much related work was available and when this manuscript could have made a greater impact on society and the journal itself. Moreover, this work is based on a full paper that was already accepted for publication; therefore, as previously mentioned, it comprises a “typical” research method, such as that of a literature review, which at the time did not comprise many articles on the subject, but included an extraction, analysis, structure, and synthesis of the content of the revised articles. This is not described in the viewpoint because it would be a repetition of work (as previously mentioned). The main aim of this viewpoint was to extract the main issues found at the time on the subject, to make the scientific community and the general public think and be aware of these crucial aspects. However, this work is still very relevant 8 months later, and readers can be referred to the full accepted paper. Please see below extracts from the mentioned work [3], with more details on the undertaken methodology, which constitutes the basis of this viewpoint. I apologize for the formatting, but it is not possible to upload tables or figures in this space, so I also attached these at the end of the new uploaded manuscript as well:

“A literature review of the research performed in this area (Jan-May, 2020) was performed. Search queries were applied to research database engines, and works were reviewed by their titles and abstracts and repeated works were excluded. From the included works, a summary includes: Type of work (article, letter, comment, etc); Database engine; Direct/Indirect consequence from COVID-19; Main subject; Goal(s); Problem raised; and Proposed solution.”

Queries used to search in the various databases and works included in the review (illustration of the methods performed in this work):

Database Query N found N included

Scopus TITLE-ABS-KEY((security OR privacy OR confidentiality) AND (covid OR pandemic)) 82 10 + 1*

Pubmed ((security OR privacy OR confidentiality) AND (covid OR pandemic)) 131 5 + 1*

Xplorer (security OR privacy OR confidentiality) AND (covid OR pandemic) 21 1

ISI TS=((security OR privacy OR confidentiality) AND (covid OR pandemic)) 33 0

ACM [[All: security] OR [All: privacy] OR [All: confidentiality]] AND [[All: covid] OR [All: pandemic]] AND [Publication Date: Last 6 Months] 9 0

TOTAL 276 1 6 + 2*

#### Anonymous [20]

Thank you very much for your comment. First of all, we want to remind the reviewer that this viewpoint was submitted in early June 2020, when not much related work was available and when this manuscript could have made a greater impact on society and the journal itself. It is therefore normal that by now (almost 8 months later), there are many more studies about similar subjects, as this is still a very critical and essential topic (this work has been available as a preprint since June 2020 [21]). Moreover, the examples given by the reviewer are not from scientific publications but from websites. Nevertheless, the authors have altered the title to a more clean, unique one by removing the word “cybersecurity,” namely, “COVID-19 and the Human Right to Privacy: A Viewpoint”. Unless the work is altered in its essence and objective, making a more specific contribution was not the main goal of this viewpoint but, again, to “expose, as well as increase awareness and discussion of COVID-19 consequences to cybersecurity and healthcare.”

## Round 3 Review:

### Further Editorial/Peer Reviewer Comments:

Dear Editor, thank you for your suggestion. Although a long time has already elapsed (7 months) since we first submitted the first manuscript of this viewpoint, we still think that the subjects discussed are as important now as they were then. However, the authors also agree that this viewpoint has the potential to propose some novel advances and perspectives that have not yet been considered and that were suggested by you. These can have more impact on the ways to address existing cybersecurity issues to foster a change in the current paradigm. Hence, this is what the authors have reviewed, within the manuscript, to consider your suggestion:

- The title has been changed to “COVID-19 and Cybersecurity: Finally, an Opportunity to Disrupt?” in order to reflect the new changes proposed by the editors.
- The Abstract was edited to include objective arguments on what needs to be done to address current problems and raise the issue of how cybersecurity needs a change in paradigm, with or without a pandemic, according to the authors’ perspectives and expertise in the area.
- At the end of the Introduction, there is a clear mention of the change of structure within the viewpoint.

Section 2, which enumerates the various cybersecurity challenges both directly and indirectly linked to the current pandemic, was left almost the same; however, for section 2.2.2, we provided a clearer description of the cybersecurity challenges (“Impact on privacy and physical integrity”). These challenges are raised in this part, and recommendations to address them are presented in a new section, “What needs changing in cybersecurity.”

Section 3 was added to enrich the viewpoint with novel ideas and perspectives to contribute to advancing the discussion of cybersecurity as a whole discipline. This section introduces the idea that humanity can go back to “normal,” but the authors stress the need for change in the way technology is thought of and how humans interact with it. Technology evolved much faster than humans could adapt in terms of how relationships and trust are established. The main perspectives provided by the authors are:

- The need for better and more adapted means to provide education and information technology literacy; moreover, now with constant mixed contexts and 24/7 web-based activities, there is not much time to process information. This needs to be more objective, intuitive and easy to grasp.
- As privacy and security are great challenges in human-computer interaction, technology design and development must focus on the pervasive line concept of all relations: trust. The authors discuss that trust needs to be properly studied and incorporated within technology development, as to date, there have been attempts to solve various pieces of the puzzle but not the real picture that needs to happen in the end. Therefore, existing solutions are clearly not enough. The authors also substantiate this perspective with recent literature supporting this subject.
- When trust is addressed, other related issues can be advanced. These include (1) better understanding of human behavior, personality traits, and victimization features, which are helpful to advance in social engineering studies; (2) extraction of trust/relation patterns, which can be useful to promote more personalized interactions as well as the identification of specific needs of users to foster more successful and trustable interactions; and (3) confident use of novel technologies such as high-fidelity digital humans as well as augmented reality to simulate the currently greatly needed contexts via videoconference (work, school, shopping, leisure, exercise, etc), with more control and integrated privacy features.

The previously provided perspectives require time and resources and may not be so quick to implement or provide adequate outcomes; therefore, the authors also suggest the development of alternative tools to easily test required cybersecurity features within technology. This can be done using anonymous simulated contexts (“digital twins”) in order to integrate usability and interaction methods to mock up interactions without the required coding (which would certainly take much longer). These digital twins can be used by anyone in the world who uses the internet, which currently is a lot of people, anonymously (to comply with legislation requirements and protect users’ privacy) but while still simulating real interactions so to easily understand the main factors that improve trust in relations and how to more quickly integrate these into technology development.

The last two paragraphs of Section 3 were maintained from the previous version.

The *Conclusion* section was also altered to reflect the changes described above.

The authors hope that with these alterations, the viewpoint is a document that can generate interest as well as have a disruptive impact.

## Round 4 Review:

Dear Editor, thank you for your reviews. Here is what the authors have changed according to your suggestions:

- Regarding suggestion 1, this was done mostly with the summarization of Section 2, as proposed in the third point below. Other important sections were changed to be more objective and to the point, such as Section 3, the Abstract, and the Conclusion. The viewpoint now has 3004 words. The authors hope that this is now in accordance with the Editor’s size requirements.
- Regarding suggestion 2, as mentioned, the first part is a summary of the already published and referenced work, but the second part is novel and has not been published anywhere. It was drafted specifically for this viewpoint according to the previous reviews by the Editor. Hence, the last paragraph of the introduction was changed to reflect just that; there is a reference to each mentioned part (eg, Section 2 for the first part of the paragraph and Section 3 for the second part). Further, to better clarify where the second part comes from, a last sentence was added: “These comprise authors’ original recommendations specific to this viewpoint.”
- Regarding suggestion 3, the authors have made Section 2 more concise and more objective, also by taking out repeated text. We hope that this section reads better and highlights the required key points.
